# Dr. Haller on the Radical Cure of Reducible Inguinal Hernia

**Published:** 1842-08-27

**Authors:** C. Haller


### ANALECTA.

***Radical Cure of Reducible Inguinal Hernia.*** By Dr. C. Haller.—Seven cases are given. The operation, as detailed in the first of these, was as follows: The patient, a man of twenty-three years, had ruptured himself in 1837. In 1840, when the operation was performed, his hernia, which was on the right side, had attained the size of a hen's egg, was soft, elastic, and contained a small knuckle of intestine. It made its appearance on the patient coughing or violently exerting himself, but receded on his assuming the horizontal position. The inguinal ring was so far enlarged as to admit the index-finger. The rectum having been emptied by a clyster, the scrotal integument was invaginated in the inguinal canal, by the index-finger of the operator; while, with the right hand, by means of the *sonde à dard* two stitches were made; the one as high as possible on the inner crus of Poupart's ligament; the other just over the angle of the two crura. A ball or cork of lint was introduced into the loop thus formed on the thread; traction was applied to one end of the thread, by which the lint-ball was carried as far as possible up the inguinal canal; the double threads were then separated, and tied down over two quills united by sticking plaster. Ice was applied to the seat of operation; the testicles were supported; the patient was ordered to bed, and put on low diet. There were slight pain and redness, but considerable swelling and hardness around the cylindrical aperture; and on the sixth day there were light febrile symptoms. As suppuration commenced from the punctures, the threads were loosened; the lint slightly withdrawn, and thus a free exit permitted to the matter. Warm applications to the part, daily ablution, and syringing of the invaginated integument, laxative clysters, constituted the treatment. The suppuration continued during fourteen days, and as it declined, the integument gradually subsided into the inguinal canal. On the 28th day from the operation, the patient, with the aid of an elastic band, could stand up, and move about a little. The inguinal canal was appreciably narrowed, and even violent coughing or straining did not reproduce the hernia. In nine months the patient dispensed with the truss. In two or three of the other cases the cure was only temporary, the hernia again descending and carrying the integument before it. But in several instances the operation has been successful.—*British and Foreign Medical Review.* July, 1842. *From Oesterr. Medicinische Jährbucher.* März, 1842.

